# Professional learning needs in using video calls identified through workshops

**DOI:** 10.1186/s12909-016-0657-6

**Published:** 2016-05-10

**Authors:** Sarah Statton, Ray Jones, Martin Thomas, Tracie North, Ruth Endacott, Adrian Frost, Dazzle Tighe, Gail Wilson

**Affiliations:** School of Nursing and Midwifery, Plymouth University, 3 Portland Villas, Plymouth, PL4 8AA UK; St Luke’s Hospice Plymouth, Plymouth, UK

**Keywords:** End-of-life, Clinical education, Learning needs, Video calls

## Abstract

**Background:**

Most people want to die at home but only half do. Supporting patients in rural locations is challenging. Video calls such as Skype, might help but are not routinely used; we should consider learning needs to increase uptake and ensure effective use. We aimed to identify learning needs of healthcare professionals (HCPs) in using video calls to support patients (and their carers) to die at home.

**Methods:**

Face-to-face workshops were held in five Southwest England locations. Participants discussed advantages, disadvantages, scenarios for use, and the learning needs of video call users. Ideas were documented on flipcharts and discussions audio-recorded. The 116 participants included nurses, allied HCPs, doctors and previously bereaved volunteers. Lists of advantages, disadvantages, scenarios and learning needs were compiled and circulated to participants. In a subsequent online workshop, 21 participants ranked seven groups of learning needs in priority order.

**Results:**

Most participants thought video calls could be used to advantage in many end-of-life scenarios, especially in rural areas. Seven themes, covering 59 learning needs for HCPs, were identified (in priority order): (i) confidence and technical ability in using video calls; (ii) being aware of how video calls fit into clinical practice; (iii) managing video calls; (iv) communication skills on ‘camera’; (v) understanding how patients and families may be affected by video call use; (vi) presenting video calls as an option to patients and families to assess their readiness; (vii) normal professional skills that become essential for effective video calls.

**Conclusions:**

Although almost ubiquitous, video call software is not routinely and effectively used in British clinical practice. Supporting patients and families at end-of-life is one example where it could be used to advantage, but clinicians need to plan and practise before using it in real situations. Learning needs were identified that could be developed into learning modules and/or courses.

**Electronic supplementary material:**

The online version of this article (doi:10.1186/s12909-016-0657-6) contains supplementary material, which is available to authorized users.

## Background

Three out of four people want to die at home [1] but nationally only half [1], and locally only 23 % [2] of people, who wanted to die at home did. The English Department of Health wants to increase the proportion of people able to die in their preferred place [3]. This mismatch between preferred and actual place of death, results from multiple and complex factors including: individual characteristics (e.g. coping skills), the illness itself, and environmental factors (e.g. healthcare professional (HCP) support) [4]. The latter are probably most influential in place of death amongst cancer patients [4]; increased homecare input and frequency of home visits are required for patients to die at home [4]. Furthermore, rurality is likely to make provision of such home support more difficult [5].

Actual place of death gives no indication on whether the death was comfortable or dignified. In the National Survey of Bereaved People (VOICES): 2014, just over half surveyed said that services definitely worked well together for patients who had spent the last three months at home, so coordination of community care can be improved [6]. Video calls could help this coordination and NHS England’s aim of seven-day access to palliative care services [7]. Hospital admission is stressful for both patients and carers during end-of-life [8] yet 20–33 % of those patients admitted may be appropriately cared for at home [9]. Supporting formal and informal carers with expert advice could help reduce crises and allow more to die at home with an improved perception of the death.

Using video calls in clinical practice is not new [10] but often expensive videoconferencing equipment is used [11]. ‘Off-the-shelf’ video calls, such as Skype and FaceTime are now ubiquitous on consumer devices and Skype is being used in chronic diseases, surgical education, clinical education, speech and language pathology, mental health, urology and pathology, but infrequently for healthcare in the home [11]. End-of-life is an appropriate situation in which to consider use of video calls as many aspects, such as efficient communication, confidentiality, empathy and multidisciplinary team working, are acute in nature and important to get right first time because there is ‘only one chance to get it right’ [12]. To ultimately improve uptake of effective use, we identified the advantages, disadvantages, scenarios, and learning needs of HCPs when using video calls to support patients in the last six months of life to die at home.

## Methods

### Design

We conducted face-to-face focus group workshops and thematically analysed the discussions. In a subsequent online workshop, participants ranked the themes identified from the face-to-face workshops. Additional file 1 gives details of the COREQ checklist.

### Setting

Five face-to-face workshops were held between May and July 2015 in locations corresponding to the main adult hospices in Cornwall and Devon. In September 2015, an online workshop was conducted inviting participants from the UK. Ethical approval was not required for this service improvement study (see Additional file 2).

### Recruitment

Three hospice befriending and bereavement volunteers were invited to work as co-authors. They helped produce short video presentations and develop the content of the workshops. Two of these (AF, DT) then helped with all the workshops. Personal contacts were used to ‘snowball’ to find people across Devon and Cornwall who might be interested to attend. A wide range of HCPs were invited to participate in the workshops (Table 1). An example of anonymised email invitation is included in the Additional file 2. Each local hospice was asked to send out leaflets (Additional file 2) informing previously bereaved volunteers of the workshops and if they wanted to participate, they were asked to contact the first author. The bereaved volunteers were people who did voluntary work for the hospice and had experienced a close bereavement.

**Table 1:** Participants in the workshops

103 healthcare professionals that participated in the face-to-face workshops	
Clinical commissioning group (4)	General practitioners (3)
District nurses (9)	Paramedics (2)
Community matrons (4)	Out-of-hours doctors (1)
Long term conditions matrons (1)	Community pharmacists (5)
Care home staff (6)	Occupational therapists (4)
Social care staff (3)	Physiotherapists (1)
Nursing agencies (2)	Speech and language therapists (3)
Community palliative care nurses (13)	Dieticians (1)
Communication skills/education Leads (5)	Chaplains (3)
Hospice social workers (3)	Healthwatch (patient participation groups) (2)
Hospice doctors (4)	Other healthcare professionals who expressed interest including medical and nursing students (17)
Hospice directors of care (2)	
Palliative care discharge team (1)	
Acute hospital staff (4)	
13 bereaved volunteers took part in the face-to-face workshops	
21 participants took part in the online workshop	
Total participants in workshops 137	

### Face-to-face workshops

We introduced the topic of video calls to participants and set the parameters for discussion. For example, we assumed current or ‘near future’ technology and although we acknowledged that internet connection was not always good in some rural areas, we made the assumption that inequity of provision was ‘another conversation’. We assumed that video calls provide more information than telephone calls but less than face-to-face visits.

Depending upon the number of participants attending each workshop, we facilitated two or three groups per session aiming for 8–12 participants per group. The same three facilitators (RJ, MT, SS) with agreed procedures were used for all workshops. The discussions focused on whether video calls could be used to support patients in the last six months of their lives to die at home. Home was classified as their own home or a care home. Each workshop had two sessions. First, participants discussed advantages, disadvantages, learning needs and research and development needs of using video calls. During this first session, previously bereaved volunteers were kept separate from HCPs to allow them to ‘settle in’ as for some group discussions like this may have been new and we also wanted to ensure that they were ready to discuss issues around end-of-life without feeling too upset about their own loss. Secondly, participants discussed scenarios, learning needs and research and development needs of using video calls. During this second session, participants were mixed to give similar balance of volunteers and HCPs in each group.

Two methods of data collection were used: (i) participants were asked to note down key points from the discussions and post them onto flipcharts under the relevant section; (ii) sessions were audio recorded. Also detailed notes were taken by one of the team members (DT) in her workshops. After each workshop the facilitators discussed whether that workshop brought up any new topics. All the notes posted onto flipcharts were typed up under the relevant sections. Audio recordings were listened to (but not transcribed) for clarification purposes. A preliminary list, with themes identified under each section, was emailed to workshop participants asking for any further comments.

### Online workshop

In a subsequent online workshop, learning needs from the face-to-face workshops were presented using seven themes. Participants were asked to rate whether or not a ‘typical’ HCP dealing with end-of-life had these skills already or would need training to develop them. Ratings were converted into scores from one (definitely yes, they have the skill so do not need to learn), through to four (definitely no, so they need to learn). Themes were then ranked in order based on the mean score.

## Results

In total, 137 people (plus five team members) participated in both face-to-face and online workshops (Table 1). Table 2 illustrates the advantages, disadvantages and scenarios of when video calls may help patients to die at home (full list in the Additional file 3).

**Table 2:** Advantages and disadvantages of using video calls to support patients to die at home

Advantages of Video calls
Reduce travel; Save time; Reduce stress for patients and families rather than attend hospital; Better access and help for remotely located patients; Good for out-of-hours; Compared to telephone call, more reassuring for some patients to see the HCP; HCP has more information for remote consultations.
Disadvantages of Video calls
Introduction of new, even simple technology becomes very difficult to understand; Video calls need to be simple to use and not add additional pressure; Possible loss of non-verbal signs; Lack of personal touch/presence; Issues of privacy changing from a telephone call to a video call; Perhaps inability to concentrate on what is being said given screen and technology; Concerns regarding security.
Scenarios
Scheduled versus unscheduled video calls; Medication queries e.g. identifying correct doses by holding up to camera; Symptoms/signs (e.g. breathing patterns), movement (e.g. exercise) – visual confirmation; Equipment problems e.g. syringe driver, nasogastric tube problems; Bringing distant family member into conversation; Case conference between family and multidisciplinary team more easily arranged; During a period of bad weather, it may be physically impossible to carry out a face-to-face visit.

Note: A full list is presented in the Additional file 3

Seven themes, including 59 learning outcomes, were identified as needing to be addressed for HCPs to use video calls effectively. All face-to-face workshops contributed towards their development although we seemed to reach ‘saturation’ with five workshops. Participants in the online workshop thought confidence and technical ability in using video calls the most important but that all seven themes need to be addressed (Table 3).

**Table 3:** Seven themes in priority order

Priority order	Mean	The seven learning need themes	Number of learning needs
1	3.26	Confidence and technical ability in using video calls.	10
2	3.17	Being aware of how video calls fit into clinical practice.	10
3	3.00	Managing video calls.	12
4	2.78	Communication skills on ‘camera’.	9
5	2.74	Understanding how patients and families may be affected by video call use.	3
6	2.61	Presenting video calls as an option to patients and families and assess their readiness.	4
7	2.48	‘Normal’ professional skills that become essential for effective video calls.	11

Note: Seven themes shown in priority order (1 equals the greatest need), under which the 59 learning needs were grouped, showing the number of learning needs for each and the mean score used to determine the priority order. The 59 learning needs are presented in full in the Additional file 3

The 59 learning needs for HCPs are presented in full in the Additional file 3 but illustrated here under the seven themes:

- **Confidence and technical ability in using video calls**: HCPs need to be confident and feel at ease in using the technology and know how to deal with technical problems. They should know how to get the best lighting, image and sound. For example, video callers should not sit in front of a window or their face will be in the shade. They need to avoid online distractions such as email alerts by, for example, setting an electronic ‘do not disturb’ sign up. They need to recognise when the technology is failing, when to revert to the telephone, and how to switch if the device allows this.
- **Being aware of how video calls fit into clinical practice**: Professionals need to know when it is best to use video calls in preference to telephone, and face-to-face in preference to video calls. They have to be aware of the implications for information governance, confidentiality, the legal position and be aware of existing NHS or Trust guidelines. They must know how to introduce video calls into care relationships. Some participants thought it was best to have face-to-face visits first with subsequent use of video calls, others thought the first contact could be a video call. If possible, video calls should be introduced in earlier stages of end-of-life and not during acute periods. HCPs must understand and plan for the practicality of using video calls as part of the healthcare system, for example, choosing whether to do a ‘video call clinic’, or mix them with other contacts. They have to be aware of the cost effectiveness and workload impact of video calls, for example whether or not it reduces cost enabling more patients to be seen.
- **Managing video calls**: HCPs should know how to find out who is within ear/camera shot at the patient’s home and be able to clarify for all participants who is included at both ends. They must be able to manage groups of people and ensure everyone can hear. For example, patients may want to speak to HCPs without particular family members present. HCPs need strategies for getting others out of the room but unlike face-to-face contacts, HCPs cannot know what is happening ‘off camera’. HCPs must know how to interpret and use the background visuals sensitively, for example, knowing what to do if they see unexpected things, which the family are not aware can be seen via the video call. They must know when to end video calls and plan follow-ups. Some follow-up face-to-face visits can last 1–2 h, but conducting video calls of such length would be very wearing on all concerned.
- **Communication skills on ‘camera’**: HCPs need to know how to use non-verbal communication during a video call. For example, they should try to maintain eye contact with the camera and if using their hands to communicate, these need to be in-shot. They need to be able to reassure with words, facial expressions, ‘virtually hand-hold’ and ‘virtually cuddle’ to offer emotional support. Also HCPs should be aware of their appearance to give an empathetic and professional contact. For example, in late night video calls, HCPs need to look professional and not stifling yawns. HCPs need to know how to deal with silence, for example, moving occasionally so that people know they are still there and the picture is not frozen.
- **Understanding how patients and families may be affected by video call use**: HCPs need to be aware that some people may ‘hold back’ emotional issues if not accustomed or not comfortable in using video calls, while it is possible that others may be more forthcoming. They need to be able to reassure users that video calls are under the family’s control and not some form of Big Brother monitoring. If applicable, HCPs need to be able to bring other family members into the video call while on site in the family home. This requires an understanding of who should be in shot for the conversation and whether or not some conversations should be private between certain members of those assembled, and how to deal with the video call during those times.
- **Presenting video calls as an option to patients and families to assess their readiness**: HCPs need to explain the advantages of video calls to patients, families, and colleagues, but understand the nuanced way in which this needs to be presented. For example, video calls could reduce footfall into a patient’s home, an advantage because it reduces disruption. However, this could be a disadvantage in that a family may feel neglected because they ‘only’ received a video call which may be seen simply as cost cutting. Hopefully video calls will make health services more efficient so that more patients can benefit from professional contact. HCPs need to assess people’s readiness to use video calls, understanding that it is not for everyone and respect patient preferences.
- **Normal professional skills that become essential for effective video calls**: HCPs need to understand the roles of other health and social care disciplines at end-of-life. For example, workshop participants thought that video calls may increase multidisciplinary teamwork aiding support and communication between team members and the family and patient, but this would only happen if HCPs already work well together. They need to be able to communicate well with people who have communication disabilities. Video calls may improve the potential of HCPs to communicate with people with hearing impairments in comparison to the telephone, but some aspects of communication may still be lost. As in all communication, HCPs need to listen to the concerns of patients and/or carers and not hear what they want to hear. They need to be able to use appropriate language for the person they are communicating with and they need to recognise when patients no longer have the capacity to make decisions or give accurate answers. HCPs need to feel confident dealing with conflict and be aware of issues of dignity and confidentiality when viewing an unconscious patient. Finally, HCPs need to know how to deal with consent, for example, if relatives have power of attorney.

## Discussion

Telemedicine and use of video calls via bespoke software within healthcare is not new [10], for example, Airedale NHS Trust uses them to support patients at end-of-life via the ‘Gold Line’ [13]. The idea of an ‘e-hospice’ has been proposed in previous work [14]. Skype-based systems are operating within healthcare, but not all of its uses have been formally studied or reported [11]. We have studied the use of video calls at end-of-life because it is a situation where quality of communication is essential for good patient care. We think this is the first study to consider learning needs of readily available and free technology such as Skype and FaceTime to support patients in the last six months of life to die at home.

Our participants thought that video calls could have a role in palliative care in the near future. This is in line with the view that telehealth within palliative care is an acceptable and feasible adjunct [15] that can facilitate an empathetic patient-HCP relationship [16]. In all but 1 of 27 studies in a recent review, the authors reported the use of Skype within clinical practice to be feasible and beneficial [11]. Despite this, at present there is little formal evidence on the clinical use of Skype in home settings [11]. The advantages and scenarios identified by our participants suggest that video calls at the end-of-life could not only offer support to allow more to die at home if this is their preferred place, but also allow HCPs to communicate with each other more effectively to coordinate patient care.

Education was seen by all our participants as important in overcoming barriers to using video calls. However, education is only one factor in the uptake of new technology. Health service providers may be slow to change, have concerns about regulation and costs, which lead to a vicious circle of inaction. Many e-health projects fail to be implemented in routine practice [17, 18]. Systematic reviews by Mair et al. [19] and Greenhalgh et al. [20] suggest that successful e-health innovation needs: (i) A shared view among users of its purpose, to understand how it affects them personally and to grasp its potential benefits; (ii) Work to engage potential users to get them to buy into the new system; (iii) Collective action to re-design heath care tasks, ensure confidence in the innovation and train participants; and (iv) Local appraisal to make best use of the innovation. Education to raise awareness of the advantages, disadvantages, and scenarios in which Skype can be used to improve the skills and self-efficacy of clinicians is a necessary but not sufficient part of this process. In addition, commissioners of services also need to consider how they might use such new technologies in a cost-effective manner.

There are limitations to this study in that we only sought the opinions of HCPs and previously bereaved volunteers in Cornwall and Devon. Although some of our participants had experience of using video calls in clinical practice and many in personal use, other regions may be less or more advanced in its use and their learning needs different as a result. Another limitation was that opinions of patients who are in the last six months of life were not sought, however we had extensive involvement from bereaved and other volunteers who well represented the patients viewpoint. We did not transcribe and formally analyse the audio tapes but used (a) the flip charts and ‘post-its’ of main points that were written by the groups as they discussed the issues, (b) detailed notes taken by one of the team members (DT) in her workshops, (c) the audio tapes when clarification or confirmation was needed. After each workshop the facilitators also discussed whether that workshop brought up any notable new topics. This is perhaps not as rigorous as transcribing each tape but we are fairly sure that we have not missed any major ideas. Two authors (RJ, SS) reviewed the data and agreed on the final list of advantages, disadvantages, scenarios, and learning needs. This list was reviewed by others in the team and was the basis of the online workshop so seems to have some face validity with this wider audience.

The 59 learning needs for HCPs presented in this report could be used to develop learning materials either some self-learning package or, more likely, some form of course. Communication skills’ training is mandatory for palliative care HCPs because it can be nurtured and improved [21]. Use of video calls in everyday life is not yet as widespread as other forms of digital communication such as email and text, but is growing rapidly [22]. The use of video calls within HCPs' day-to-day work is even less frequent. Many undergraduate nursing students and registered nurses feel anxious and emotionally distressed when faced with providing care to a patient at the end-of-life [23, 24]. Therefore, training in the form of a learning module and/or course will ensure they feel empowered to incorporate this into their work meaning they can focus on the patient and not the technology.

We envisage that new learning needs and scenarios will become apparent as the technology is used more widely. However, the learning needs identified here will serve as a good starting point. These learning needs may diminish as people start to use it in their everyday lives; nevertheless many organisations still run professional development courses in the use of telephone [25].

We present detailed learning needs but further work is needed to design suitable learning resources and we are currently considering how to take this forward. For example, our seven themes could be condensed into four learning outcomes: (i) HCP introduces the idea of video calls; (ii) HCP preparation before the video call; (iii) HCP conducts the video call; (IV) HCP reflects on the video call and the information gained or not. Jelle van Gurp et al. [16] have devised an implementation guide for palliative homecare by means of a video call. The findings presented here in this study have been used to expand upon the implementation guide proposed [16] (Additional file 4) and this could form the framework of the learning module. A next step will be to (i) liaise with commissioners and service providers about piloting the technology and how it should be included in contracts, and (ii) develop modules and/or courses incorporating these learning needs.

## Conclusions

Although almost ubiquitous, video call software is not routinely and effectively used in British clinical practice. Supporting patients and families at end-of-life is one example where it could be used to advantage, but clinicians need to plan and practise before using it in real situations. We have identified areas 59 learning needs in seven themes. Further work is needed to design suitable learning resources. The seven themes may be condensed into four groups focussing on introducing, preparing, conducting, and reflecting on the video call.

## Ethics

The study was funded by Health Education South West Innovation Fund. As an educational innovation and service improvement study it did not require approval from a Research Ethics Committee. Further information on this is given in the Additional file 2.

## Additional files

Additional file 1: COREQ checklist. (DOCX 19 kb) Additional file 2: Further information on ethical considerations, (ii) recruitment email for health professionals (DOCX 26 kb), and (iii) recruitment for bereaved volunteers (PDF 184 kb). (ZIP 192 kb) Additional file 3: Further information on advantages, disadvantages, scenarios and professional learning needs. (DOCX 38 kb) Additional file 4: A step-by-step implementation guide to health professionals. (DOCX 22 kb)
