# Supplementary material for: Professional learning needs in using video calls identified through workshops
Source: BMC Med Educ. 2016 May 10;16:140. doi: 10.1186/s12909-016-0657-6 (PMC4863338; doi:10.1186/s12909-016-0657-6)
Supplement: Additional file 1: — COREQ checklist. (DOCX 19 kb) [file 12909_2016_657_MOESM1_ESM.docx]

Additional File 1

**COREQ checklist**

1. Interviewer/facilitator Which author/s conducted the interview or focus group?

Ray Jones, Sarah Statton, Martin Thomas

1. Credentials What were the researcher's credentials? E.g. PhD, MD

PhD, MBChB, and MSc respectively

1. Occupation What was their occupation at the time of the study?

Professor Health Informatics; Research Assistant; Palliative Care Nurse respectively

1. Gender Was the researcher male or female?

Male; Female; Male

1. Experience and training What experience or training did the researcher have?

RJ had been either active researcher or supervisor of well over 100 mixed methods research studies over his research career of 38 years.

SS had undertaken research modules in her medical degree and had 2-3 years post qualification clinical work experience.

MT is a very experienced Specialist Palliative Care Nurse who completed a Masters project based on focus groups and interviews.

1. Relationship established Was a relationship established prior to study commencement?

All participants had been in email correspondence and/or telephone conversations with SS.

1. Participant knowledge of the interviewer What did the participants know about the researcher? e.g. personal goals, reasons for doing the research

All partiicpants had been given written information as part of the recruitment process. This included information about the researchers, the funding, and the purpose of the project.

1. Interviewer characteristics What characteristics were reported about the interviewer/facilitator? e.g. Bias, assumptions, reasons and interests in the research topic.

The motivation for the project was described in the introduction/background to the paper.

1. Methodological orientation and Theory What methodological orientation was stated to underpin the study? e.g. grounded theory, discourse analysis, ethnography, phenomenology, content analysis

Grounded theory.

1. Sampling How were participants selected? e.g. purposive, convenience, consecutive, snowball

Snowball

1. Method of approach How were participants approached? e.g. face-to-face, telephone, mail, email

Usually email with occasional telephone.

1. Sample size How many participants were in the study?

116 in face-face workshops and 21 in online workshop.

1. Non-participation How many people refused to participate or dropped out? Approximately 30 people did not turn up for the workshops mainly as a result of other commitments.
2. Setting of data collection Where was the data collected? e.g. home, clinic, workplace

In five face-face workshops held in meeting rooms in four hospices and one university in Devon and Cornwall.

1. Presence of non-participants Was anyone else present besides the participants and researchers?

No

1. Description of sample What are the important characteristics of the sample? e.g. demographic data, date

The occupations (including bereaved volunteer) are described in the paper.

1. Interview guide Were questions, prompts, guides provided by the authors? Was it pilot tested?

Yes.

1. Repeat interviews Were repeat interviews carried out? If yes, how many?

No. These were focus groups conducted with five separate groups.

1. Audio/visual recording Did the research use audio or visual recording to collect the data?

Yes. We used ‘flip charts’ on which we collected ‘post-its’ or wrote comments. We also audio recorded (two recorders for security) each session. And in some sessions (see paper) extensive notes were written by one of the participants.

1. Field notes Were field notes made during and/or after the interview or focus group?

Yes. See above. The researchers also met after each workshop and reflected upon how each session went and whether it produced new findings.

1. Duration What was the duration of the interviews or focus group?

We started with lunch for participants to get to know each other, then an introduction to the purpose of the afternoon, then session 1 (1 hour), a tea break, then session 2 (1 hour).

1. Data saturation Was data saturation discussed?

Yes. See paper.

1. Transcripts returned Were transcripts returned to participants for comment and/or correction?

No. We did not transcribe the tapes. All participants were invited to take part in a subsequent online workshop and at the end all participants were sent a summary of results.

1. Number of data coders How many data coders coded the data?

Two – SS and RJ

1. Description of the coding tree Did authors provide a description of the coding tree?

No.

1. Derivation of themes Were themes identified in advance or derived from the data?

Derived from the data.

1. Software What software, if applicable, was used to manage the data?

Done ‘by hand’ (just using wordprocessor)

1. Participant checking Did participants provide feedback on the findings?

Participants in the online workshop rated the importance of each of the 7 identified themes.

1. Quotations presented Were participant quotations presented to illustrate the themes / findings? Was each quotation identified? e.g. participant number.

We did not use quotations in the paper.

1. Data and findings consistent Was there consistency between the data presented and the findings?

Yes.

1. Clarity of major themes Were major themes clearly presented in the findings?

Yes.

1. Clarity of minor themes Is there a description of diverse cases or discussion of minor themes?

Yes
