# Supplementary material for: Professional learning needs in using video calls identified through workshops
Source: BMC Med Educ. 2016 May 10;16:140. doi: 10.1186/s12909-016-0657-6 (PMC4863338; doi:10.1186/s12909-016-0657-6)
Supplement: Additional file 2: — Further information on ethical considerations, (ii) recruitment email for health professionals (DOCX 26 kb), and (iii) recruitment for bereaved volunteers (PDF 184 kb). (ZIP 192 kb) [file 12909_2016_657_MOESM2_ESM.zip › Additional File 2 ethics and recruitment.docx]

**Professional learning needs in using video calls to support end-of-life care at home.**

**Additional File 2**

**Ethical Considerations**

The authors themselves deemed that ethics committee approval was not needed as (a) the funding source for this project was Health Education South West Innovation Fund [1]. This call clearly indicated that projects should be service improvement and the timescale for funding and project completion did not allow for the (usually lengthy) time required to apply for and obtain ethics committee funding; (b) the Heath Research Ethics Authority states [2] that ethical approval is not required for “Quality assurance and quality improvement studies, program evaluation activities, performance reviews, and testing within normal educational requirements if there is no research question involved (used exclusively for assessment, management or improvement purposes).”; (c) universities, including our own, regularly involve a range of stakeholders, including patients, in developing new curricula.

The box below shows an anonymised example of email correspondence recruiting participants:

Dear XX

**Developing the education needs of healthcare professionals, patients and carers to use video calls to support End of Life Care in the patient’s home.**

I would like to invite you to attend a workshop at ____ on _____ 2015 in __________. The session will last from midday until 4pm. There will be a light lunch provided and tea or coffee available throughout the afternoon. We will pay for your travel expenses.

We think that video calls, such as Skype or Facetime, may be a useful support tool for patients, families and carers in end of life care at home, but we cannot be sure.

I have attached a leaflet, which provides more information about the research, how it will be organised, how we will use the information and the potential future impact of the research.

If you are able to attend please can you confirm by the _____ (date) via email: [sarah.statton@plymouth.ac.uk](mailto:sarah.statton@plymouth.ac.uk). If, however, you are not able to attend this session but are able to attend one of the other four face-to-face workshops or the online workshop (see dates in the enclosed leaflet) then please let me know. Please feel free to contact us for further information about the research project.

We will issue all healthcare professionals who attend with a Plymouth University certificate of attendance as you may wish to count this day towards your Continuing Professional Development portfolio.

Yours sincerely

Sarah Statton on behalf of

Professor Ray Jones, Plymouth University

Martin Thomas, St Luke’s Hospice

References

[1] Health Education South West Membership Council Innovation Fund 2014/15 <http://www.devonlmc.org/?sc=libext&id=25533&acc=10800476>

[2] Health Research Ethics Authority (2016). Does your study require ethics review. <http://www.hrea.ca/Ethics-Review-Required.aspx>
