# Supplementary material for: Professional learning needs in using video calls identified through workshops
Source: BMC Med Educ. 2016 May 10;16:140. doi: 10.1186/s12909-016-0657-6 (PMC4863338; doi:10.1186/s12909-016-0657-6)
Supplement: Additional file 2: — Further information on ethical considerations, (ii) recruitment email for health professionals (DOCX 26 kb), and (iii) recruitment for bereaved volunteers (PDF 184 kb). (ZIP 192 kb) [file 12909_2016_657_MOESM2_ESM.zip › Additional File 2a Leaflet for bereaved volunteers.pdf]

We think that providing better contact via video calls, such as through Skype or Facetime, may allow healthcare professionals to support families and patients in end of life care, but we cannot be sure.

What do you think? We would like to hear your opinion on this topic.

For more information about the research project, please contact:

sarah.statton@plymouth.ac.uk

or

Dr Sarah Statton
4 Portland Villas
School of Nursing and Midwifery
Plymouth University
Drake Circus
Plymouth
Devon
PL4 8AA

PLYMOUTH UNIVERSITY

St Luke's Hospice Plymouth

Developing the education needs of healthcare professionals, patients and carers to use video calls to support End of Life Care in the patient's home.

The above project is being funded by Health Education South West.

Background:

Previous research has revealed that three out of four people want to die in their own home[1]. However, out of all the deaths in Cornwall and Devon in 2012 only 23% did so[2].

Our Aim:

We are holding five face-to-face workshops with 22 healthcare professionals and 10 bereaved relatives at each workshop. We want to explore their opinions on video calls to support a patient to die in their own home. Also we hope to explore what training requirements all parties involved would need.

How it will work:

Each workshop will comprise an initial welcome and explanation, some sub-group discussions in groups of six/seven and a final summary. Discussions will be recorded and the views expressed analysed afterwards. A report will be produced detailing the findings and circulated to all the participants for their approval after the session. It is important to note that individual responses will not be attributable (ie, you will be anonymous)..
Subsequently we will run an online workshop, which may be further developed into an online CPD module for end of life care.

Where are the workshops?

- *Date*, *time* at Barnstaple
- *Date*, *time* at Exeter
- *Date*, *time* at Plymouth
- *Date*, *time* at St Austell
- *Date*, *time* at Torbay
- Thursday 3rd September 2015 4-5pm online workshop

There will be a light lunch provided and tea or coffee available throughout the meeting.

We will pay for your travel expenses (except for the online workshop!)

And then…

The findings from all the workshops will be used to write a final report at the end of October 2015. We hope to publish the findings of the research project. Moreover, we hope to build on the findings of this research and show that video calls can offer reassurance and prevent some of the acute hospital admissions in palliative care patients. Thus, allowing more people to die at home as they wished.

Do you think you would be able and willing to attend? To help you consider your involvement, these are the sort of questions that we might be discussing:

1. During your recent bereavement were there times when you felt you needed the help and support of a healthcare professional for your loved one?
2. Did you get the support for your loved one? If so, how did you get support:
   a. Phoned a healthcare professional for advice e.g. GP, district nurse, palliative care nurse, hospital doctor.
   b. Arranged for a healthcare professional to visit your loved one in their own home.
   c. Made an appointment for your loved one at a GP surgery, hospital etc.
   d. Other support.
3. Do you use the internet?
4. Do you use Skype or Facetime?
5. Do you think a video call from a healthcare professional could have offered you the support and help your loved one needed?
6. What do you think professionals and families need to ‘learn’ or consider to be able to make use of this technology effectively?
