# Supplementary material for: Professional learning needs in using video calls identified through workshops
Source: BMC Med Educ. 2016 May 10;16:140. doi: 10.1186/s12909-016-0657-6 (PMC4863338; doi:10.1186/s12909-016-0657-6)
Supplement: Additional file 3: — Further information on advantages, disadvantages, scenarios and professional learning needs. (DOCX 38 kb) [file 12909_2016_657_MOESM3_ESM.docx]

**Professional learning needs in using video calls to support end-of-life care at home.**

**Additional File 3**

**Advantages of video calls to support a patient to die at home:**

**Immediate advantages (compared to telephone calls):**

1. **Clinical decisions.** The ability to see patients, their facial expressions and other movement provides healthcare professionals more information than just audio, and may increase the quality of the assessment, leading to a better outcome for the patient. Sometimes patients do not tell healthcare professionals how unwell they actually are, and seeing them helps healthcare professionals notice improvement/deterioration in the patient. A video call provides more information, especially with unknown patients, and can help to prioritise the chain of response e.g. who needs a home visit now. Also a video call could help to prioritise calls during out-of-hours. A video call allows an assessment of the environment and can prepare the healthcare professional for a home visit (although many argue that video calls should follow not precede home visits).
2. **Improves communication for people with communication disabilities.** For example those who are hard of hearing, struggle with their speech or struggle with manual dexterity in dialling the numbers on a telephone. Video calls can enhance communication using two senses – sight and hearing. Also there is the possibility of typing a message at the same time.
3. **Feeling of connection.** Seeing someone as well as talking to them may help reduce any feeling of isolation for the patient.
4. **More likely to pick up safeguarding issues.** Video call provides more information about the home environment meaning a healthcare professional can make better, less error prone, decisions.

**Consequent advantages if video calls substitute for hospital/practice visits:**

1. **Patients may be more likely to give a fuller history.** Video calls may provide more information than hospital or practice visits because patients will be more relaxed at home.
2. **May prevent unnecessary hospital admissions.** Out-of-hours it may offer reassurance to both health care professional and patients and family, so preventing urgent hospital admission.
3. **Reduced stress, cost, and time for patients, particularly those who live in remote/rural locations.** For example, the patient may be too unwell to travel to appointments. Also there is the added worry and stress of travelling to the appointment, finding somewhere to park, time spent in the waiting room etc. a video call also saves patient and family costs.

**Consequent advantages if video calls substitute for home visits by healthcare professionals:**

1. **Keeps healthcare professionals accessible at all times.** During times of adverse weather, healthcare professionals may struggle to make home visits to patients. Video calls could make healthcare professionals more accessible.
2. **Reduces footfall into patients’ homes.** For example the use of a video call could reduce the footfall into the patient’s home.
3. **Maintain independence in own home for the patient.** A video call may allow a patient to remain in control of their illness. Thus making it less ‘medicalised’.
4. **Saves the healthcare professional time and travel costs and enables more patients to be seen.**
5. **Improves healthcare professional safety in a lone working environment**.

**Consequent advantages if video calls allow multiple participants:**

Although audio conference call facilities are available, the more widespread and normalized use of multiple participant video calls may make various types of ‘conference call’ more frequently used.

1. **Ability to involve relevant others who may be many miles away.** For example a conference call could be arranged. Thus reducing the amount of communication one family member would then have to repeat back to the family. Instead they are all involved in key decisions, meaning things can progress quicker, an overall benefit for the patient.
2. **Multidisciplinary teamwork.** A video call can be made into a conference call, so multiple healthcare professionals can be involved. Thus saving time and travel for all involved, whilst ensuring a quicker and better outcome for the patient.

**Longer term impacts:**

1. **Video calls instead of telephone calls may enhance bereavement support services.** The emotional and psychological support via a video call may minimise prolonged bereavement.

**Disadvantages of video calls to support a patient to die at home:**

**Compared to face-to-face contact:**

1. **Less opportunity for emotional support.** Video calls provide less information and prevent touch compared to face-to-face contact. So providing emotional or psychological support is more difficult. It may be more difficult to form a relationship with someone.
2. **No opportunity to act physically.** A video call does not allow the healthcare professional to be opportunistic in comparison to face-to-face consultation. For example if a problem occurs then the healthcare professional cannot do anything physically to help. Also, not able to do ‘hands on work’, such as that required by a speech and language therapist.
3. **Insufficient visual or other information.** Even though they may have a video image of the patient, if the heath care professional does not know the patient it is still going to be difficult to spot deterioration and healthcare professionals will tend to air on the side of caution, compared to face-to-face. Smells (such as urine) are not part of a video call but can be very important as to how well the patient is coping.

**Compared to telephone call:**

1. **Unfamiliarity and poor use of the technology.** Healthcare professionals may come across as ‘stilted’ in a video call. For example, the position of the camera may not be correct, body language can be misconstrued, and it can be difficult to get eye to eye contact. Healthcare professionals may be distracted by ‘other things’ happening e.g. emails popping up on screen.
2. **Video calls can be more intrusive for patients than telephone calls**. For example concern was raised that as patients become more unwell, they may not want to be seen or to see themselves during video calls. They may also be embarrassed if they are no longer able to maintain their home and this is visualised during video calls. Video calls may be an invasion of their privacy.
3. **Confidentiality**. Telephone calls are more usually ‘contained’ via the handset. Video calls can also be contained via headphones or a handset but more typically users have sound on ‘loudspeaker’. This may raise issues of confidentiality: who else is present in the room with the patient? Who else is with the healthcare professional?
4. **Security**. Some IT specialists have concerns about the security of video calls (more than the confidentiality of telephone calls). What is the risk (benefit to hacker) to try to hack into a video call?
5. **Multiple participants.** If (see advantages) video calls are more likely to facilitate multiple participants, the healthcare professional may not be able to track everyone present at the same time e.g. may not notice if someone has become upset/distressed.

**Compared to telephone and face-to-face contact:**

1. **Unintentional disclosure.** Compared to a telephone call where callers are usually aware of what can be heard by the person being called, and a face-to-face visit where it is clear that everything can be ‘seen’, unfamiliarity and lack of skills with video calls may mean that calls reveal visual ‘things’ that neither recipient wants to see. For example the patient may reveal inappropriate body parts via the video call. Similarly, healthcare professionals may reveal things in the ‘background’ such as other patients’ information, posters, or other healthcare professionals.
2. **Unintentional increase in inequalities.** If video calls are used by healthcare professionals in place of face-to-face contact it may unintentionally disadvantage certain groups of individuals. For example people with a hearing impairment may have advantage (over telephone) by being able to see lip movement, but may be disadvantaged (compared to face-to-face) if they are unable to effectively lip read through video calls. Patients with mild forms of dementia may struggle to use/know how to use video call equipment and this may not be recognised by healthcare professionals.
3. **Unintentional consent issues.** For example if the patient lacks capacity and the healthcare professional is video calling a relative/carer, but they turn the camera towards the patient.
4. **More risks for healthcare professionals**. Healthcare professionals will not have all the same information available compared to face-to-face consultation, which could lead to errors being made if there were a sense of ‘false reassurance’ from the video call. A video call may lead the healthcare professional to make inappropriate snap judgments.

**Consequent disadvantages on health service organisation:**

1. **Limitations on health service resources may lead to video calls replacing face-to-face contacts.** Although video calls may be better than no contact at all, and probably better than telephone calls, they are not as good as face-to-face contacts. There was concern that introduction of video calls will lead to diversion of resources and a poorer service. (There was discussion about the sequence of how/when to introduce use of video calls).
2. **Possible resource misallocation.** Greater use of video calls may be more efficient, allowing fewer healthcare professionals to support more patients, saving which can be used elsewhere in the NHS (advantage) but this may lead to job loss and political choice to reduce resources (disadvantage).
3. **Scalability and equity of service provision**. Video calls for people at end of life will only realistically be available to those who already have wifi, equipment, and sufficient skills. This may mean that resources can be re-allocated to ensure face-to-face contacts for others but during the upscaling of such services it may lead to worsening of inequalities of provision.

**Existing problems not improved by video calls:**

1. **Continuity of care may not be improved.** Although having video calls is another option to face-to-face contact and telephone calls, it does not guarantee that every time patient’s video call they will speak to healthcare professionals that know them. Even though healthcare professionals can see patients via video calls, it is still very hard to recognise deterioration or change if the healthcare professional does not know the patient and the medical records are not extremely good quality.
2. **Persistent video calls.** Although it seems unlikely, there is perhaps some risk that some patients may – like the telephone – make many calls and ‘abuse the system’.

**Uncertainties:**

1. **The legal aspects surrounding giving advice via video calls.** What is the legal status of healthcare professional decisions, if an error is made, when made by video call compared to face-to-face or by telephone?

**Implementation issues:**

1. **Problem of adoption.** Patients, carers, and healthcare professionals may be reluctant/resistant to the introduction of video calls into end of life care.
2. **It is unlikely that the NHS would use video calls with IT novices.** Educating patients to exercise ‘new’ skills during a time of great distress is going to be very difficult. Video calls will be very difficult to adopt ‘from scratch’ if patients have rapid diagnosis and very short prognosis. It is very unlikely to be cost effective for healthcare professionals to train patients in the use of video calls. So (see inequalities) video calls are only likely to be used by those who already know how to, or can easily get help from family, friends, or volunteers, and have access to the Internet in their home**.**
3. **Location of healthcare professional in video calling.** Many healthcare professionals already have problems with confidential phone calls given the propensity to shared office space. The location for the video call needs to be appropriate so that the patient sees an appropriate background and does not hear ‘interference’ from a call centre environment.

**Scenarios when video calls may be used to support patients to die at home:**

1. **Scheduled versus unscheduled video calls.** There are four different situations when a video call could be used:
   - 1. In hours and scheduled.
     2. In hours emergency.
     3. Out-of-hours and scheduled.
     4. Out-of-hours emergency.
2. **Medication queries could be addressed via a video call.** For example identifying correct doses of medications by holding up the packet to the camera.
3. **The breathing pattern of a patient could be observed via a video call.** This could apply to the breathing associated with end of life, the long term condition chronic obstructive pulmonary disease (COPD) or an acute change, such as during an infection.
4. **During a period of bad weather it may be physically impossible to carry out a face-to-face visit.**
5. **Physiotherapist/occupational therapist could use a video call.** For example to demonstrate exercises or check the patient performing the exercises correctly. Also to check if the patient is coping/knows how to use a piece of equipment (especially if just been delivered).
6. **Nasogastric tube problems could be addressed via a video call.** For example it is easier to talk through how to unblock a nasogastric tube if able to see what type they are using or if able to give a demonstration via a video call. This may avoid a trip to hospital. A video call would allow for visual confirmation of the type of feed and how administered; an error may be detected, which would have been missed otherwise.
7. **Syringe driver problems could be addressed via a video call.**
8. **If a drain moves and blood comes out around the access point.** A competent family member could be talked through via a video call how to re-dress it. Instead of a visit to hospital.
9. **A video call could offer support to a healthcare professional that is on site with the patient.** For example supporting the assessment and decision making of the paramedic crew (especially regarding treatment escalation plans and preferred place of death questions).
10. **Carer’s group via a video call e.g. meet virtually for coffee and cake.** Speaking to other people in similar situations may not only offer support but help to reduce loneliness.

**59 learning needs for healthcare professionals to use video calls to support patients to die at home:**

**Confidence and technical ability in using video calls:**

1. **Know how to get the best lighting, image and sound.**  For example, callers should not sit in front of a window or their face will be in the shade. They need light falling on to their face.
2. **Know when to use headphones and be able to advise the patient or family about their use.**
3. **Know how to use the video call ‘test function’ to check how you look and sound.**
4. **(May need to) Be able to type at the same time as video call to make best use of their time and not to forget important items/actions from the contact.**
5. **Be aware (and probably turn off) online distractions such as email alerts while using video call.**
6. **Be able to manage the background to the camera and video call.** For example, making sure that you have a physical and electronic ‘do not disturb’ sign up.
7. **Recognise when the technology is failing and they need to revert to telephone.**
8. **Be confident in using the technology, how to deal with technical problems, and need to feel at ease.**
9. **Know how to switch between telephone and video call – if the devices do not allow this, so perhaps need to know how to schedule a video call.**
10. **Understand and be able to use different video call systems (Skype, Facetime, other) to adapt to patients.**

**Being aware of how video calls fit into clinical practice:**

1. **Know how to ensure that the patient’s wishes always come first.**
2. **Be aware of their legal position.** Compared to a telephone or face-to-face contact particularly if a wrong decision is made, and know when to recommend that a face-to-face consultation is needed. For example, some people claim that commercial video call services are not ‘secure networks’ and should not be used (but no such discussions are had about telephone, mobile systems, fax). Nevertheless the healthcare professional needs to know local and legal position.
3. **Know when it is best to use video call in preference to telephone and face-to-face in preference to video call.**

- **Be aware of any differences between telephone, video call, and face-to-face with respect to information governance and confidentiality.**
- **Know how to introduce video call into a care relationship.** This might mean having a first visit face-to-face with subsequent use of video calls, and/or introducing use of video calls in earlier stages of end of life and not necessarily during the acute stage. On the other hand some participants suggested that video calls might be used before a face-to-face visit as it ‘puts a face to the voice before the healthcare professional comes to the door’.

1. **Be aware of any NHS or Trust guidelines that exist for use of video calls.**
2. **Understand and plan for the practicality of using video calls as part of the system.** For example whether to do all the video calls as a ‘video call clinic’, or just mix in with other contacts. Need to know how to deal with lots of waiting video calls.
3. **Be aware of the cost effectiveness and workload impact of video calls**. For example, whether or not it reduces cost enabling more patients to be seen but how this might impact on overall workloads or manager’s expectations. Healthcare professionals need to make sure that ‘the system’ does not have unrealistic expectations so creating more stress for staff and subsequent deterioration in patient care.
4. **Know how to document a video call.** This is likely to be written in to the medical record, but in the longer term some form of recording (with consent) may be feasible and preferred. But the healthcare professional needs to know how to extract main points.
5. **Understand how video call might link to other systems and innovations.** For example, how might it link to TEP (treatment escalation plan) and patient shared records?
6. **Knowing not to mix personal and professional usernames for video calls.** Healthcare professionals need a professional video call username.
7. **Understand the differences between telephone, video call, and face-to-face and be able to decide which is best for the patient and family.**

**Managing video calls:**

1. **Know how to find out who is within ear/camera shot at the patient’s home and be able to clarify for all on the call who is included in the video call at both ends.**
2. **Know how and when to reassure the patient or family if there is a time delay, pixellation, or freezing of the picture.**
3. **Know when during a telephone call it is appropriate to try to switch to a video call.** For example where an image would enhance the call e.g. pain management, breathing.
4. **Be able to invite patient and family to switch from telephone to video call without being too ‘pushy’ recognizing that some people don’t want to be seen.**
5. **Be able to direct a family or professional carer to put the patient ‘in shot’ or move the camera.** Need to be sure of how and which direction to move, up/down left/right in/out etc.
6. **Being able to manage the group of people at home on the video call given that everyone can hear.** For example, it is possible that a patient wants to speak to the healthcare professional without a particular family member present. The healthcare professional needs to somehow realise this and have a strategy for getting others out of the room. On the other hand the healthcare professional cannot see all family members at same time and there may be things happening off camera such as people in distress. The healthcare professional needs strategies for knowing (if necessary) what else is happening in the room.
7. **Being able to manage the position of the video call - camera/sound.** For example, the healthcare professional might want an onsite healthcare professional or family member to move away from the bedside to talk.
8. **Knowing what to do if the healthcare professional has witnessed unexpected or illegal objects and activities, which the patient/family was not aware can be seen via the video call.**
9. **Knowing when to end a video call and plan a follow up call.** Some follow-up face-to-face visits can be 1-2 hours, conducting this as a video call may be very wearing on patient and family.
10. **Be aware that colours via a video call will depend on lighting and may not be true to real life.** This may be important if assessing for example, the colour of skin, vomit etc. So the healthcare professional may need to ask the family to try variations in lighting.
11. **Know how to effectively end a video call, i.e. to say goodbye.**
12. **How to manage a case conference by video call**. Know how to decide who will chair the case conference and ensure that the person has appropriate chairing skills. (Who chairs will depend on the relationships between healthcare professionals, collaboration and context).

**Communication skills on ‘camera’:**

1. **Know how to interpret and use the ‘background’ visuals in a video call in a sensitive way.** For example, in a video call there may be potential to pick up on hazards in the room or poor use of equipment or other concerns in the room. In a face-to-face situation it would easier to have a ‘slightly longer look’ to reach a decision if some issue needed to be raised with the family carer, and this could be done in a friendly and concerned manner. On a video call the healthcare professional will have to ask the carer to focus the camera on the item of concern and then explain why the healthcare professional wanted to look at that. (This difficulty may be reduced with some form of ‘robotic control telepresence’ such as a Kubi).
2. **Know how to use non-verbal communication via video call that is not misleading and improves the communication.** For example, the healthcare professional should try to maintain eye contact as much as possible with ‘the camera’ and if the healthcare professional uses their hands to communicate these need to be ‘in-shot’.
3. **Be able to give reassurance to patients and families simply by words and facial expressions.** They need to be able to ‘virtually hand-hold’ and ‘virtually cuddle’.
4. **Be aware of their appearance, facial expressions and background to give the patient an empathetic and professional contact.** For example, overnight video calls the healthcare professional needs to look professional and not (at 2am) stifling yawns.
5. **Know how to deal with silence on the video call, healthcare professional needs to move a little so that patient or family know they are still ‘there’ (and picture not frozen).**
6. **Be able to be ‘normal’ in front of the camera and not become ‘camera shy’.**
7. **Be able to ensure that both the healthcare professional and the family and others in the home are able to concentrate on the call and not on the technology.**
8. **Be able to carry out other tasks without detriment to visual cues.** For example, it might be necessary to complete a proforma or medical record during the video call. This needs to be done without appearing to lose interest in the patient or family by looking down to type.
9. **Know how to demonstrate exercise or movement techniques (e.g. inhaler technique) visually or to assess similar movement using ‘wider angle’ on camera**.

**Understanding of how patients and families may be affected by video call use:**

1. **Be aware that some patients or family members may ‘hold back’ emotional issues if not accustomed or not comfortable in using video calls, while it is possible that others may be more forthcoming using video calls.**
2. **Be able to reassure families that video calls are under the families control and not some form of Big Brother monitoring.**
3. **Be able to handle bringing another family member into a video call while on site in the family home.** This requires an understanding of who should be ‘in shot’ for the conversation and whether or not some conversations should be private between certain members of those assembled, and how to deal with the video call during those times. For example, some family members may feel excluded if they are not in the camera shot, but someone has to hold the device and the microphone needs to be near the current speaker for good sound.

**Presenting video calls as an option to patients and families and assess their readiness:**

1. **Be able to explain the advantages of using a video call but understand the nuanced way in which these should be presented to families and other professionals.** For example, use of a video call could reduce footfall into the patient’s home. This can be an advantage by reducing disruption to the family but may produce some disadvantages in that healthcare professionals may spot ‘incidental’ needs that will not be seen on a video call. Some families may feel neglected by only video call contact. So healthcare professional has to be able to sell the advantages while acknowledging concerns. Many families and patients may see video calls simply as ‘cost cutting’. Hopefully it will save the NHS money but at the same time it means that healthcare professionals can have contact and give advice more often with limited resources, as well being more timely and convenient for families.
2. **Be able to counter the tabloid press scare stories and present security and other concerns in a proportionate manner.**
3. **Knowing how to assess what stage people are at e.g. readiness to use video calls.**
4. **Understand that video calls are not for everyone and to respect patient and family preferences.**

**‘Normal’ healthcare professional skills that becomes essential for effective video calls:**

1. **Understand the roles of other health and social care disciplines at end of life.** Participants thought that video calls may increase multidisciplinary teamwork aiding support and communication between team members and the family and patient, but this only happens if healthcare professionals work well together anyway.
2. **Understand and be able to communicate well with people who have communication disability.** Participants thought that video calls may improve the potential of healthcare professionals to communicate with people with communication disabilities (compared to the telephone) but some aspects of communication may still be lost by a video call. Healthcare professionals need these skills anyway but video call may ‘stretch’ their abilities compared to face-to-face. For example, motor neurone disease patients may only have non-verbal communication remaining.
3. **Recognise when a patient no longer has the capacity to make decisions or give accurate answers.** This is no different than in a face-to-face situation but the healthcare professional may lose some non-verbal cues and also then has to negotiate the hand-over of the video call link back to a family carer, and to have a continued conversation remembering that the patient is likely still to be there even if not seen.
4. **Be able to deal with conflict** **while conducting a video call.**
5. **Be aware of issues of dignity and confidentiality when viewing an unconscious patient at the request of the family carer.**
6. **Be aware of how to deal with consent.** For example if a relative has power of attorney.
7. **Know how to speak slowly and clearly showing lip movement for the hard of hearing.**
8. **Know how to ensure that the patient and family have given consent.** This is an ‘enhanced version’ of what is needed on the telephone.
9. **Be aware that different family members may have different levels of knowledge and may or may not have come to terms with the situation.** This is presented in a more acute manner to the healthcare professional when in a ‘group’ video call with family members present who would not be present in a telephone call, and is more difficult to control than when face-to-face.
10. **Be able to give emotional and psychological support using a video call to promote the principles of a good death.** Support to minimise prolonged bereavement.
11. **Have good communication skills for telephone and face-to-face and not forget these on the video call.** For example, the healthcare professional might want to recap/summarise what been said, needs good listening skills, be interested and compassionate listening.
