# Supplementary material for: Professional learning needs in using video calls identified through workshops
Source: BMC Med Educ. 2016 May 10;16:140. doi: 10.1186/s12909-016-0657-6 (PMC4863338; doi:10.1186/s12909-016-0657-6)
Supplement: Additional file 4: — A step-by-step implementation guide to health professionals. (DOCX 22 kb) [file 12909_2016_657_MOESM4_ESM.docx]

**Professional learning needs in using video calls to support end-of-life care at home.**

**Additional File 4**

| **Healthcare professional introduces the idea of a video call:** | |
| --- | --- |
| **Step 1** | Assess a patient’s readiness to use a video call in the first face-to-face consultation. |
| **Step 2** | Understand when it is best to use a video call in preference to the telephone and face-to-face in preference to video calls – individual to the patient’s needs at that time. |
| **Healthcare professional preparation before the video call:** | |
| **Step 3** | The context: private consultation room with a ‘do not disturb’ sign; turn off any other distractions such as a bleep; be on time. |
| **Step 4** | Non-verbal communication: position the camera for best lighting and check the sound quality. Then focus on the patient and not on your own presentation; let yourself be absorbed into the conversation and provide personal attention and emotional contact. |
| **Healthcare professional conducts the video call to the patient/carer:** | |
| **Step 5** | Gain consent of patient/carer and check who else is present. |
| **Step 6** | Communication: listen, be patient, show empathy and let everyone have their say. |
| **Step 7** | Address discrepancies between what has been told and what you see. Do not hold back from sensitive topics. |
| **Step 8** | Agree a plan of action and arrange the next contact. Do not be afraid to seek the advice of other health and social care professionals and communicate this to the patient/carer. |
| **Step 9** | End the video call and document in the patient’s records. |
| **Healthcare professional reflects on the video call to the patient/carer:** | |
| **Step 10** | Reflection upon the information gained or not gained from the video call and any lessons learnt for future video calls. |

**Table 1: A step-by-step implementation guide for healthcare professionals to provide palliative homecare by means of a video call. Jelle van Gurp et al^16^ implementation guide has been expanded upon using the results from this study.**
